# Interference Level Detector with PCB HDI Rogowski coil for PLC Narrow-Band Applications

**DOI:** 10.3390/s23156682

**Published:** 2023-07-26

**Authors:** Aleksander Lisowiec, Marcin Habrych, Pawel Michalski, Bogdan Miedzinski, Grzegorz Wisniewski

**Affiliations:** 1 Łukasiewicz Research Network—Tele & Radio Research Institute, Ratuszowa St. 11, 03-450 Warsaw, Poland; aleksander.lisowiec@itr.lukasiewicz.gov.pl (A.L.); pawel.michalski@itr.lukasiewicz.gov.pl (P.M.); 2 Faculty of Electrical Engineering, Wroclaw University of Science and Technology, Wybrzeze Wyspianskiego St. 27, 50-370 Wroclaw, Poland; bogdan.miedzinski@pwr.edu.pl (B.M.); grzegorz.wisniewski@pwr.edu.pl (G.W.)

**Keywords:** narrow-band PLC transmission, supraharmonics, interference level detector, Rogowski coil

## Abstract

This article presents and discusses the structure, principle of operation, and operational properties of a newly developed interference level detector (ILD) designed to measure conducted supraharmonic disturbances (1–150 kHz) in the power grid and to assess the effectiveness of narrow-band Power Line Communication (PLC) transmission, especially in the PRIME technology. The usability assessment was made on the basis of the validation and the results of tests carried out in a low-voltage network with non-linear loads. Appropriate practical conclusions were then formulated.

## 1. Introduction

There has been increasing interest recently in using power grids for data transmission, both low- and high-voltage, of a common structure and topology. This is known as Power Line Communication technology (PLC) [1,2]. According to the recommendation of international standards, low- (2–150 kHz) and high-frequency bandwidth (2–32 MHz) can be used [3]. When it comes to low-voltage networks, a low-frequency bandwidth is most often applied in most European countries, as specified by the European Committee for Electrotechnical Standardization (CENELEC) [4]. The main task of power grids is the transmission and distribution of electrical energy. Therefore, additionally superimposed signals should not have any negative impact on the quality of the transmitted 50/60 Hz electricity. However, electrical phenomena occurring during the grid’s operation may significantly interfere with the transmission capacity, particularly transient events. These are mostly due to the operation of non-linear receivers or short circuits and/or switching. It is obvious that the electrical parameters of the power grid are far from those required for effective high-frequency signal transmission. Attenuation, in particular, has the greatest impact, resulting in significant, undesirable, unexpected increasing and decreasing variations of the Signal-to-Noise Ratio (SNR) value with time [5]. Therefore, the effective use of PLC transmission over any electric power network needs online control of the quality of the transmitted signal as well as control of the noise introduced into the communication channel (both induced and conducted). High-frequency perturbations of both current and voltage within the used bandwidth (i.e., from 3 kHz to 150 kHz) are commonly introduced into the power grid, mainly due to the operation of numerous non-linear loads (e.g., modern light sources), commutator motors, and all kinds of pulse rectifiers and frequency converters. It should be noted that most digital modulations of the electrical signal result in interference [6,7]. In practice, the voltage response is usually measured to control the level of interference in transmission channels [8,9]. However, the conducted interferences can be basically rated by analysis of the current detector measurement data. They are of great importance for evaluating the impact of receivers powered from the grid, especially non-linear ones, on the efficiency of narrow-band PLC transmission. However, to evaluate a low-current, high-frequency signal, a dedicated measuring transformer and/or transducer is needed. These are usually made from a low-loss core made of special amorphous and/or nanocrystalline material appropriately formed magnetically [10,11]. Such structures are inconvenient in practice due to their sensitivity to temperature and/or mechanical stress. Therefore, they must be protected by using stable mounting hardware on the controlled cable. This applies in particular to two-piece cores clamped on the cable line (such as magnetic couplers in Broadband over Power Line (BPL) PLC systems [12]). Moreover, with the increase in the dimensions of the power cable, the cross-section of the magnetic core, and thus, its weight, increases. Therefore, one needs to pay particular attention to how they are installed. The authors, basing their work on the results of previous experience, have found that for current signals over 5 mA within the low-band frequency range (3–95 kHz), the detector based on the Rogowski coil made with high-density interconnect printed circuit board technology (PCB HDI) with a transformation ratio (sensitivity) around 1 mV/A (for 50 Hz) meets the expectations. The Rogowski coil’s additional advantage is that its sensitivity increases with frequency; therefore, it is equal to 1.9 V/A at 95 kHz. Therefore, an appropriately designed structure of a current interference level detector has been developed and fully laboratory tested under a specified, modeled field environment, which is a novelty. It must be mentioned that the results of the first attempts to solve this problem were presented in [13]. They were focused primarily on the selection of the structure and geometrical dimensions of the Rogowski coil to meet the requirements of the measuring system. However, it was found that this model needed to be respectively modified to be immune to externally induced random disturbances, and the user’s expectations concerning the interface were not met. This was due to the fact that the displayed data did not provide sufficient information about the conducted current perturbations being measured.

This paper presents the concept, structure, and research results of the developed interference level detector, specially dedicated to the low bandwidth (3–95 kHz) range based on the Rogowski coil made with PCB HDI technology. It discusses its performance under interference due to conducted current of 5–51 mA at narrow-band frequencies from 1 kHz up to 150 kHz, respectively. The study of PLC communication (in PRIME technology) and signal detection efficiency was carried out for randomly distorted currents as well as voltage waveforms due to linear and/or non-linear electrical energy receivers fed from the grid. The appropriate conclusions and practical recommendations were formulated on the basis of the research results.

### 1.1. Literature Review

The continuous increase in the application of power electronics technologies, particularly those associated with renewable energy systems (RES), has led to the emergence of emissions not in the classical frequency range (subharmonics, high harmonics, inter-harmonics), called supraharmonics (SH) [9]. SH emission is a new phenomenon and can be characterized as a harmonic distortion with a frequency range from 2 kHz to 150 kHz [9]. The presence of SH can significantly affect PLC communication. It also has a detrimental effect on distribution systems in general, as well as the usability of electrical equipment. It causes, among other things, overheating of power transformers or loss of smart metering communication [9]. From the point of view of low-frequency PLC transmission, knowing the harmonic content in the grid in the 2–150 kHz range is very important. Unfortunately, currently, there is no suitable measuring equipment available on the market for this purpose. Moreover, although the literature on the subject presents some measurement results, there is no information about the performance characteristics of the measurement system [9]. For example [14], presents the design of a measurement system that can be used for studies involving SH emission in the frequency range of 2–150 kHz in real grid scenarios. It also discusses the commissioning and network combination in the real grid used to study these emissions. A 4-channel acquisition system with two Rogowski coil sensors that measure the same current was employed. Although the authors do not provide the construction parameters of the Rogowski coils, their bandwidth suggests that they are either coils with a magnetic core or/and very small geometrical dimensions, which makes it impossible to use them on power cables. In addition, the authors report that the system was validated for frequencies up to 2.5 kHz. A comparison of measurement methods for current and voltage distortions in low-voltage networks in the frequency range from 2 to 150 kHz (SH) is presented in [15]. The comparison encompasses the methods informatively described in the IEC (International Electrotechnical Commission) and CISPR (Comité International Spécial des Perturbations Radioélectriques) international standards, as well as other innovative techniques presented in the literature. All these methods are based on voltage signaling. The possibility of using new amorphous-nanocrystalline alloys with improved temperature stability in a variety of applications is presented in [16]. These alloys give some hope for their effective use as magnetic cores of current sensors for new measuring apparatus for SH emission measurement. However, this requires the development of appropriate meters and their validation under field conditions. A discussion of the main issues of harmonics at the moment and in the near future is presented in [17]. It underlines the impact of harmonics, inter-harmonics, and SH on protection and metering. It states the need for new and improved standards and points out the measurement and data analysis issues. The authors in [18] study the existing power quality disturbances and identify SH emissions as a field of interest. They also present the design and initial tests conducted on a measurement system for SH. The parameters and configurations to be performed during the measurements in the smart grid platform are also discussed. On the basis of the analysis of the current state of knowledge, it can be concluded that there is a lack of appropriate measurement equipment for higher frequencies, especially for SH. Therefore, developing tools for easy and accurate measurement of SH emissions in the power grid are essentially needed. Although the main purpose of this article is to demonstrate the usefulness of the developed ILD for assessing the effectiveness of low-frequency PLC transmission, the authors see the possibility of its wider use, e.g., to study the size of power losses and the heating rate of power transformers, especially dry ones used in mines.

### 1.2. Contributions

The contributions of this article can be summarized as follows.

- Based on the Rogowski coil, appropriately designed and constructed with PCB HDI technology, the concept of the measuring system intended for e-management of detection and measurement of current harmonics with a frequency range from 2 kHz to 150 kHz was developed. Since the interference level detector is intended for use both in low-power and high-power grids, the geometric dimensions of the Rogowski coil have been carefully chosen so that it is possible to perform measurements in wires (cables) with a diameter of up to 0.05 m, which are the most common in such grids. The construction of the coil, made with PCB technology, was a compromise with respect to the value of the transformation ratio, equal to 1 mV/A (at 50 Hz), and the effective measurement frequency bandwidth, which was extended up to 150 kHz.
- Validation was performed for a harmonics current of 5 mA within the range of 1 kHz-150 kHz, respectively. The study of the PLC communication in a low-voltage power grid (in PRIME technology) and signal detection efficiency was carried out for randomly distorted currents as well as voltage waveforms due to linear and/or non-linear electrical energy receivers being fed from the grid.

The paper is organized as follows. Section 2 presents the structure and assumed operational parameters of the interference level detector. Section 3 discusses the method and scope of validation together with the results of laboratory tests. Section 4 is devoted to testing the efficiency of the detector system under simulated real working conditions of PLC transmission in the low-voltage network. It presents research results and discussions. Finally, Section 5 concludes the paper.

## 2. Structure of the Assumed Design and Operational Parameters of the Detector

### 2.1. Construction of the Detector and the Principle of Operation

**Figure 1:**
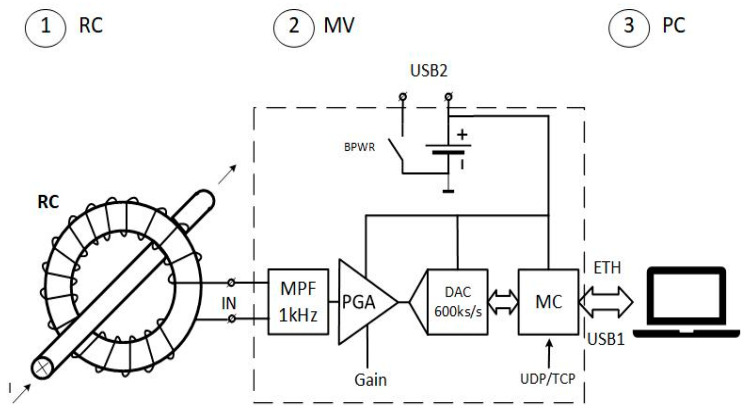
Schematic diagram of the interference level detector; 1—Rogowski coil (RC), 2—measuring system (MV), 3—computer (PC); (HPF—high pass filter; PGA—programmable gain amplifier; DAC—digital/analog converter; MC—microcontroller; BPWR—switch for battery charger).

**Figure 2:**
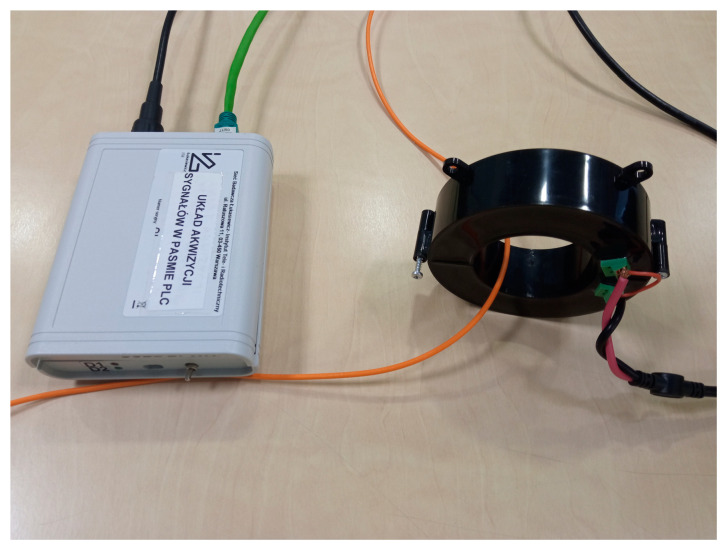
The interference level detector layout view.

The key element of the detector is a Rogowski coil. Therefore, its frequency characteristic should be selected and matched properly to ensure the highest possible sensitivity of the measurements for the required dimensions of the so-called transformer internal window [13]. The window should enable the detector to be located properly onto the 1-phase low-voltage conductor/cable of the size used in practice. It should be noted that it is difficult to meet high sensitivity requirements without the use of a special magnetically formed core material. However, as the authors found, this difficulty can be overcome in practical applications by the use of the Rogowski coil made with PCB HDI technology [19]. Moreover, the Rogowski coil made with this technology with evenly distributed secondary winding (printed circuit boards) over the coil periphery displays relatively high immunity to external interfering electromagnetic fields. Additionally, its sensitivity depends very little on the location of the primary conductor inside the internal coil window [20,21]. Therefore, the use of the Rogowski coil (particularly made with PCB HDI technology) as a detector of conducted current perturbations is fully justified. A simplified block diagram and layout view of the developed interference detector is shown in Figure 1 and Figure 2. It consists of three basic blocs, 1—the Rogowski coil (RC) (superimposed on a conductor with a controlled current), 2—the measuring system (MV), and 3—the computer (PC) (with the “PLC acquisition” application installed). USB1 is a port for uploading a binary file with appropriate settings, whereas USB2 is used as a battery charging port.

The measured input signal from the Rogowski coil is fed (in the measuring system) to a high-pass filter (HPF) with a cut-off frequency of 1 kHz. The signal is then sent via a differential amplifier (PGA) with adjustable gain to a 16-bit SAR analog-to-digital converter and further to the W7500 module (containing an ARM microcontroller) via the Ethernet link (ETH) to the computer (PC). The sampling frequency of the SAR converter is equal to 483 kHz, which, in principle, enables measuring signals with frequency content up to 241.5 kHz, but the measurement bandwidth of the developed detector was intentionally limited to 150 kHz, as this was the frequency range of interest. The measuring system (MV) can be powered externally through one of the two ports. However, during measurements, it should be powered only from the internal battery, and no cables should be connected to the USB1 and USB2 ports (neither to the computer nor to an external power source). In this mode of operation, the level of measurement data interference is the lowest.

### 2.2. Advantages over Other Solutions

The developed ILD is easy to deploy in real-life operations due to several factors. The Rogowski coil sensor is a splittable type (Figure 2), which makes it possible to install it on an electric cable without making any disconnections. This feature makes the developed ILD stand out against other devices made for the same purpose. The ILD device is powered by an internal battery during operation, which makes it independent of any external power sources. The connection of the ILD device to the PC computer is via an Ethernet cable, which is quite resilient to external interferences.

## 3. Method and Scope of Validation

**Figure 3:**
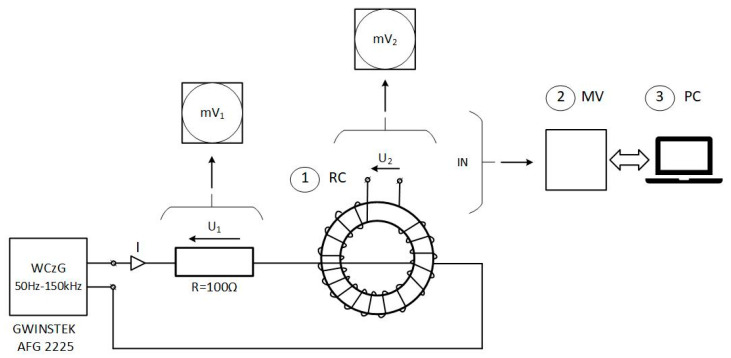
Block diagram of an electric system for validation (WCzG—frequency generator; mV_1_, mV_2_—digital multimeters 4150; RC (1)—Rogowski coil, MV (2)—measuring system of the detector, PC (3)—computer, R—standard resistive shunt class 0.01, 100 Ω).

In order to validate the performance of the developed detector, appropriate tests were carried out using the arranged electric system, the sketch of which is shown in Figure 3.

**Figure 4:**
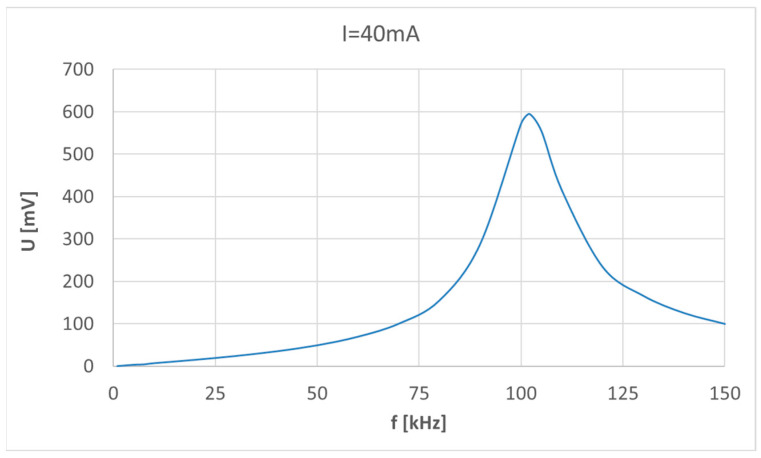
Amplitude-frequency response of the Rogowski coil for 40 mA.

The value of the current (0.1–50 mA) in the circuit with adjustable frequency (1–150 kHz provided by the signal generator GWINSTEK AFG 2225) was controlled by monitoring the voltage drop *U*_1_ across the 100 Ω class 0.01 resistive shunt. In the first step, the amplitude-frequency response of the Rogowski coil (*U*_2_ = f(*f*)), Figure 4, was measured for the coil disconnected from the MV system. It can be seen that the largest gain is for a frequency slightly higher than 100 kHz.

The coil’s amplitude-frequency characteristic, stored in a text file, is then recalculated after being entered into the detector microprocessor control circuit upon the activation of the “PLC acquisition” application. The recalculation consists in approximating it by spliced polynomials of 3 degrees in the range from 0 Hz to 241.5 kHz. After connecting the Rogowski coil to the MV measurement system, the response of the interference level detector to the measured input current signals in the range from 0.1mA to 50mA and frequencies from 1 kHz to 100 kHz was controlled and recorded. The accuracy of the measuring instruments was around ±0.05%.

The frequency spectrum Y(*f*) of the Rogowski coil’s primary current was determined with the use of Discrete Fourier Transform from the samples of the coil’s output voltage according to the equation:

(1) $$y\left(j\right)=\sum_{k=0}^{n-1}x\left(k\right)\cdot e^{\frac{-I\cdot 2\cdot \pi \cdot j\cdot k}{n}}$$

where *y*(*j*) is the *j*th spectral line, *x*(*k*) is the *k*-sample of the coil output voltage, *I* is the unit imaginary number, and *n* is the number of samples acquired. The sample acquisition interval was equal to 0.1 s, so the frequency resolution was equal to 10 Hz. The spectrum displayed on the computer screen was the Y(*f*) spectrum corrected each time by the inverse amplitude frequency characteristic of the coil, *A*(*f*). The correction consisted in multiplying each spectral line *y*(*j*) computed from (1) by the correction coefficient *cc*(*j*),

(2) $$cc\left(j\right)=\frac{1}{A(\frac{f}{j\cdot 10\;Hz})}$$

The ILD also has the option of turning off the above-described correction of the Y(*f*) spectrum. In this mode of operation, it can be used to measure the amplitude-frequency characteristic of the coil.

Additional spectral correction, removing to some extent the spectrum leakage, can be carried out (optionally) by the mean-square operation of summing closely spaced spectral lines. In the corrected spectrum, each such closely spaced group of spectral lines with indexes from *p* to *q* is substituted by a group of spectral lines of the same cardinality where only one spectral line *y*(*f_c_*), with frequency *f_c_*, is different from zero. The frequency *f_c_* and the amplitude of *y*(*f_c_*) are computed according to the following equations:

(3) $$f_{c}=\left\lfloor \frac{\sum_{j=p}^{q}j\cdot {\left(y(j)\right)}^{2}}{\sum_{j=p}^{q}{\left(y(j)\right)}^{2}}\right\rfloor \cdot 10\;Hz$$

(4) $$y\left(\frac{f_{c}}{10\;Hz}\right)=\sqrt{\sum_{j=p}^{q}{\left(y(j)\right)}^{2}}$$

**Figure 5:**
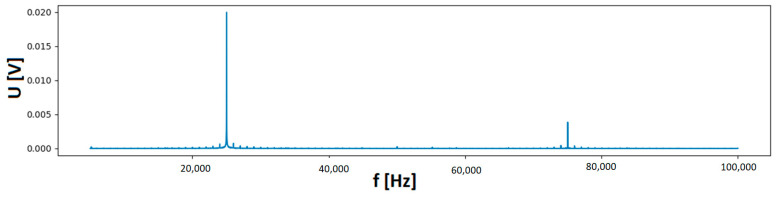
Image of the detector’s response to an input interfering current of 45mA and frequency of 25 kHz.

For the highest gain of the MV system, the measured range is limited to around 30 mV at the output of the Rogowski coil. An output spectral line image for the input current value of 45 mA, at a single frequency equal to 25 kHz is shown as an example in Figure 5 (another spectral line of lower amplitude and frequency around 75 kHz visible in the figure is due to waveform deformation of the supply voltage during the test) (1).

**Figure 6:**
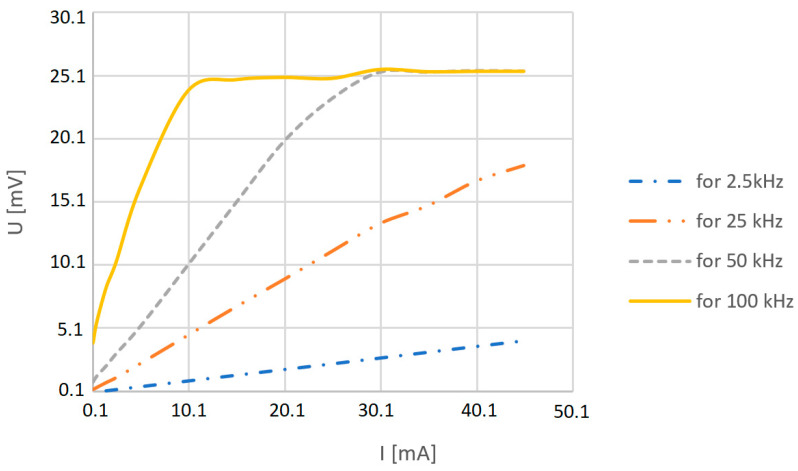
Output spectrum line voltage *U* value versus interfering current *I* (0–45 mA) and its frequency (2.5 kHz–100 kHz).

The output voltage measurement results obtained by the detector show a practically linear correlation between the value of the Rogowski coil output voltage spectral line amplitude and the current amplitude at a given frequency (Figure 6).

**Figure 7:**
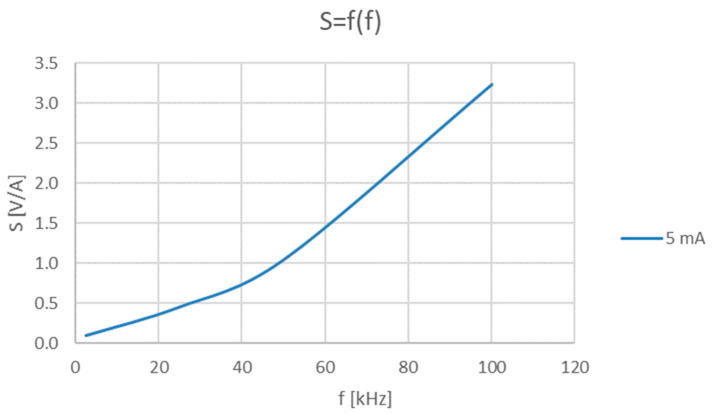
Transformation ratio (S) versus frequency of 5mA current interference signal.

However, the slope of the voltage–current relationship depends on the frequency and is determined by the amplitude-frequency characteristic of the Rogowski coil. The electrical equivalent circuit of the coil is a series resonance circuit made of the coil self-inductance and inter-winding capacitance [19]. Below the resonance frequency of this series resonance circuit, at frequencies up to 50 kHz, the coil’s output voltage increases linearly with frequency (at constant current amplitude), which is a demonstration of the Faraday law. At frequencies above 50 kHz, the series resonance circuit starts to modify the coil’s output voltage amplitude. The calculated transformation ratio *S* (Figure 7) shows that a 2-fold increase in the frequency below 50 kHz (e.g., from 20 kHz to 40 kHz) increases the value of the *S* 2 times, whereas, above 50 kHz (e.g., from 50 kHz to 100 kHz), the S increases 3 times.

Therefore, the sensitivity (over 0.1 mA) of the measurement increases with the interfering frequency, which is convenient for the effective detection of supraharmonics, particularly at higher frequencies. It should be noted that the spectrum can be displayed in both voltage and current values resulting from the transformation ratio of the Rogowski coil.

## 4. Investigation of Interference in the Low-Voltage Network with PLC Transmission

### 4.1. Sources of Interference of Narrow-Band PLC Transmission in the Low-Voltage Power Grid

**Figure 8:**
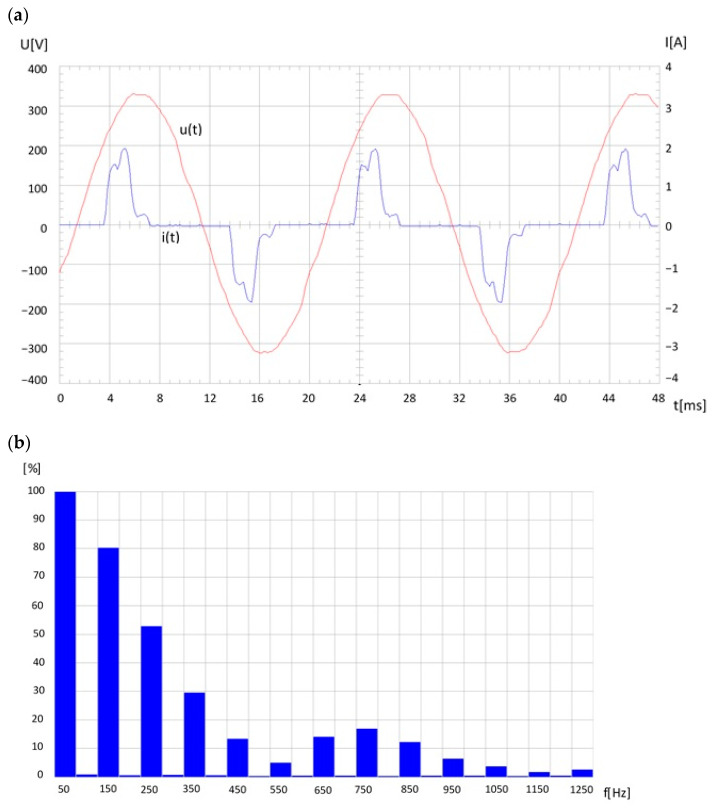
Voltage and current waveforms in a low-voltage grid due to a group (7) of fluorescent lamps (CF) (**a**), current spectrum (**b**).

The main threat to effective narrow-band PLC transmission in power grids is the interference generated primarily by any digital (non-linear) electronic device working in the impulse mode and/or the use of switching power supplies. Regardless of the PLC transmission, this interference also significantly deteriorates the power quality indices in the grid. Large interference is introduced by any fluorescent light source (CFL) with electronic ballast. An example of the energy quality deterioration in a low-voltage grid, especially the current, due to a group (7) of compact fluorescent lamps, is shown in Figure 8.

**Figure 9:**
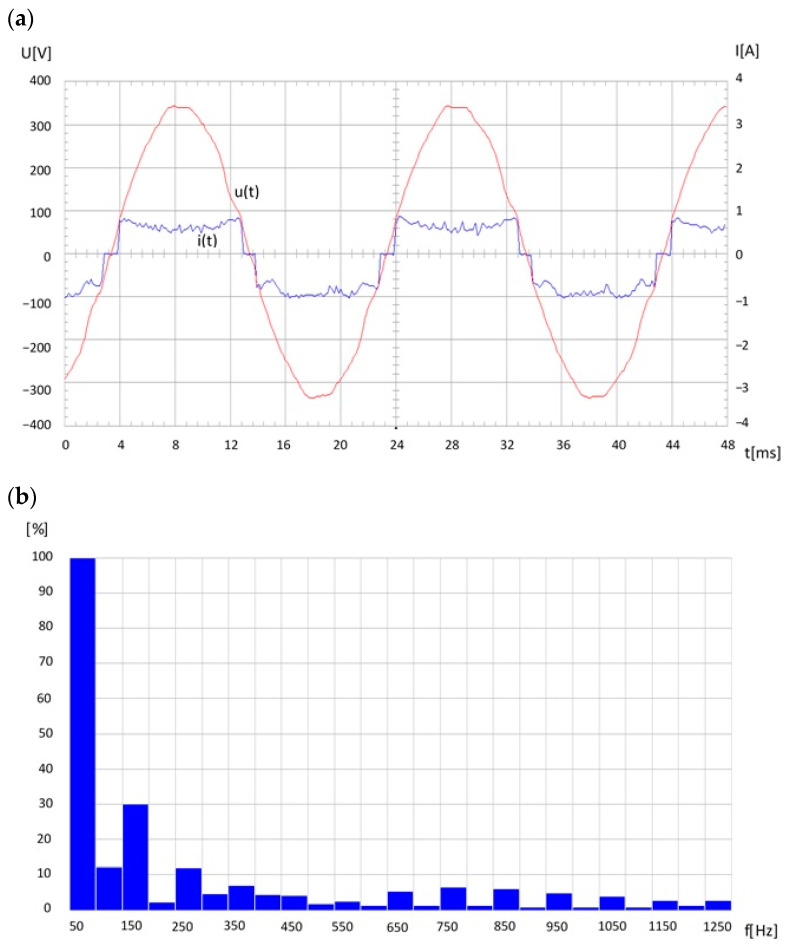
Voltage and current waveform due to 1-phase adapter powering a LED light source (**a**), (**b**) current spectrum.

Switched-mode power supplies also have a similar impact on the network, shown, for example, for a 1-phase adapter powering a LED light source, Figure 9. It should be noted that in the case of the 3-phase UPS (Uninterruptible Power Supply), particularly for server room use, different distortions of current and voltage waveforms in different phases as well as in the neutral conductor can be found.

**Figure 10:**
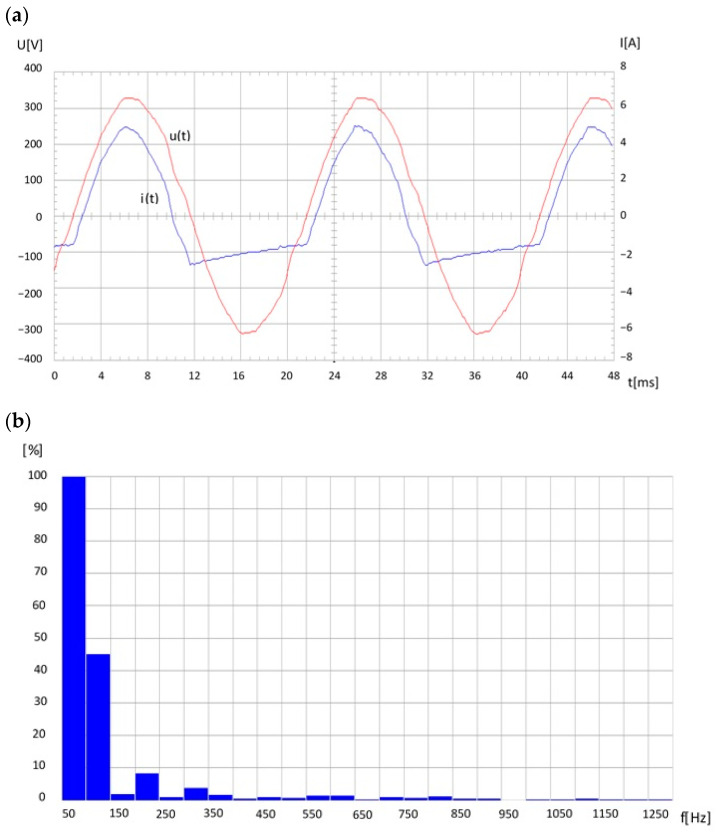
Voltage and current waveform deformation due to a hair dryer (**a**), (**b**) current spectrum.

The operation of all types of commutator electric motors (used, e.g., in home appliances) also creates a threat to the quality of the electricity supply (see Figure 10) and PLC transmission.

However, power quality measurement devices are not adapted to measure supra-harmonics, and therefore, they are not able to detect high-frequency disturbances. Their frequency range is, in practice, limited to the 40th harmonic.

**Figure 11:**
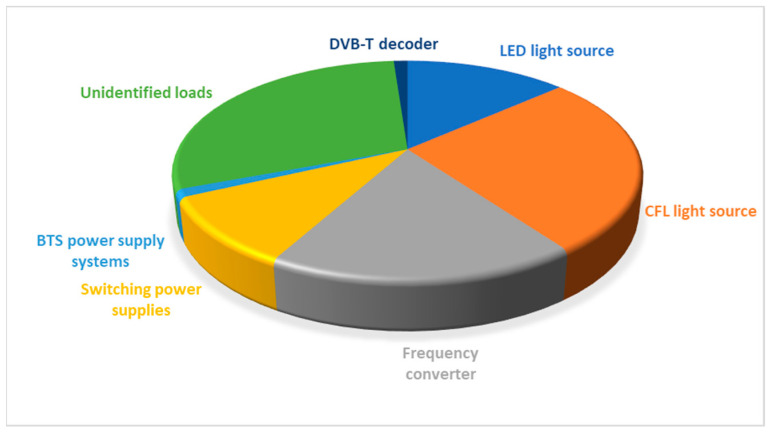
Share of end-user loads in the identified 260 interference sources in OSGP PLC transmission.

The conducted research on the identification of 260 sources of interference at end-user loads of narrow-band PLC communication in the Open Smart Grid Protocol (OSGP) band (75 and 86 kHz), based on the voltage response, showed that the main contributions are made by CFL light sources, frequency converters, light emitting diodes (LED) light sources, switching power supplies, base transceiver stations (BTS) power supply systems, and DVB-T decoders. The share of the identified loads can be compared to the share of the so-far unidentified loads, as shown in Figure 11 [7].

### 4.2. Purpose and Scope of Research

**Figure 12:**
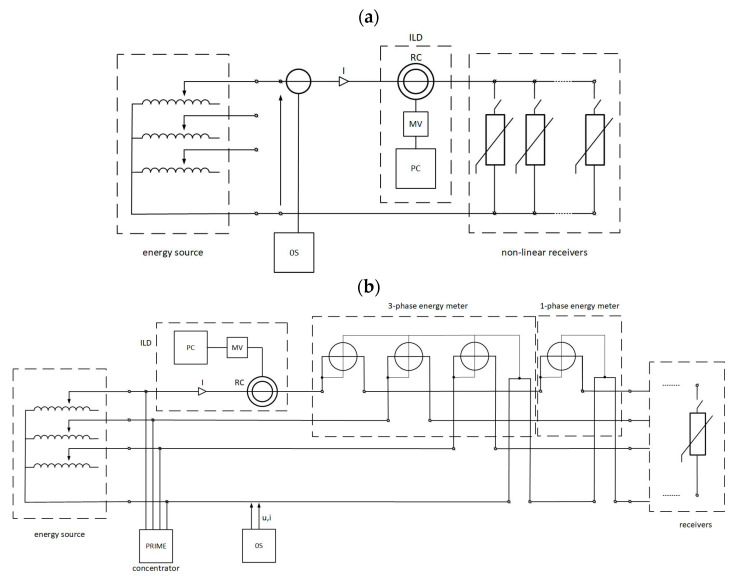
Schematic diagram of the measuring electric system for testing the efficiency of the developed interference level detector (ILD); (**a**)—measurement of interferences due to particular receivers, (**b**)—interference in PLC PRIME transmission technology.

The aim of this research was to test the usefulness of the developed interference level detector (ILD) for the quality control of narrow-band PLC transmission in the 3–95 kHz band. The interference introduced by selected (non-linear) low-voltage receivers, which primarily deteriorate the quality of electricity supply, was taken into account. The tests were carried out in the measurement system, the diagram of which is shown in Figure 12. It was performed for different levels of waveform deformation, especially current waveforms, and thus for different levels of conducted disturbances. The impact of interferences on PLC transmission in powerline intelligent metering evolution (PRIME) technology (42–89 kHz) was evaluated as an example.

**Figure 13:**
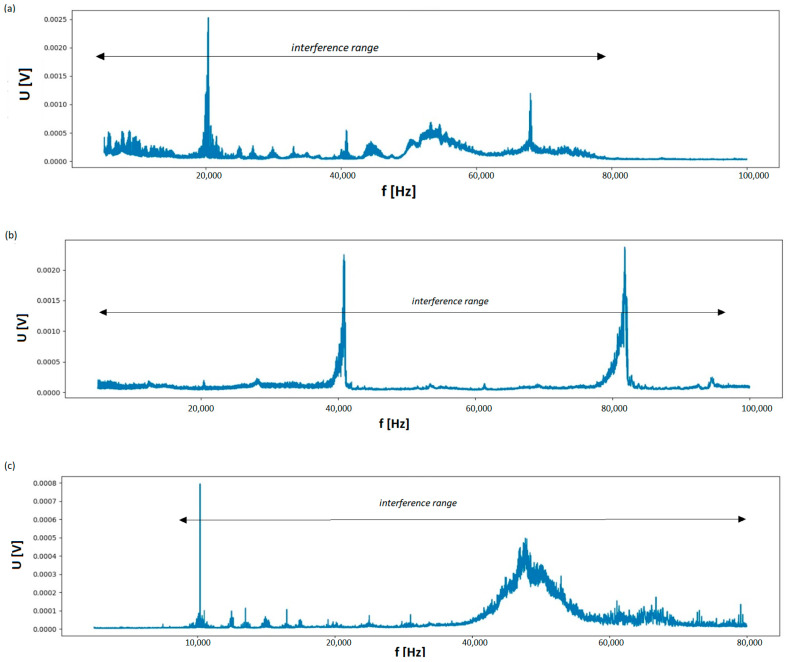
Interference spectrum generated by the selected low-voltage non-linear electricity receivers. (**a**)—group (7 different types) of fluorescent lamps; (**b**)—1 phase adapter of a LED light source, (**c**)—hair dryer (with commutator motor).

The conducted tests showed that all selected non-linear receivers are possible sources of interference within the examined frequency range. However, the spectrum of the disturbances may differ from each other. This applies to both the range and their maximum value, compared in Figure 13. For example, the interference generated by fluorescent lamps ranges from about 3 kHz to about 80 kHz. The maximum values of disturbing voltage pulses (from 0.5 mV to about 2.5 mV) appear at frequencies of 20 kHz, 40 kHz, 55 kHz, and about 70 kHz, respectively (see Figure 13a). Taking into account the amplitude-frequency characteristics of the Rogowski coil being used, it can be appropriately converted into current value if needed. 

On the other hand, the interference generated by the single-phase LED impulse adapter covers the entire tested frequency range from about 3 kHz to 100 kHz, but its maximum values (about 2 mV) occur regularly at 40 kHz and at about 83 kHz, respectively (see Figure 13b). The spectrum due to the hair dryer, equipped with a commutator motor, is characterized by an average pulse at a frequency of about 68 kHz and a value of up to about 0.4 mV, but with a much larger spectral width of around 10 kHz. The short impulse with a frequency of around 20 kHz visible in Figure 13c does not result from the operation of the dryer but most likely is due to disturbance introduced from the external power grid (city side), which is not galvanically isolated from the one in the lab. It should be noted that such impulses happened randomly during the entire testing period. Having completed the research on the nature and amount of interference due to selected receivers, the usefulness of the ILD detector for quality control and immunity to the interference of PLC transmission in the PRIME technology was checked. It was carried out in the measurement system shown in Figure 12b.

**Figure 14:**
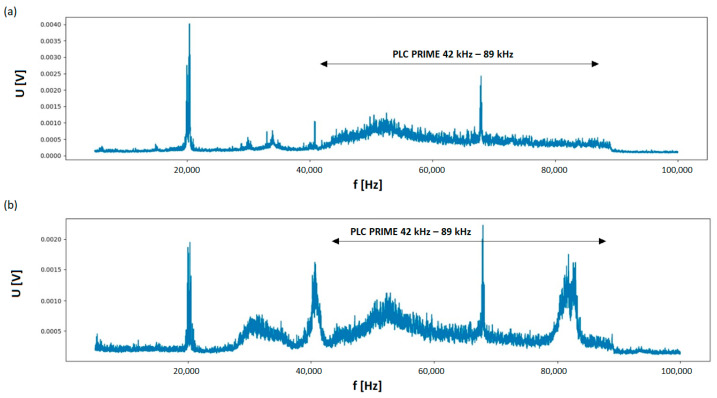
The spectrum of the signal transmitted in the PRIME technology in a circuit without interference (**a**) and under interference due to the operation of non-linear receivers (a set of 7 fluorescent lamps plus an adapter of a LED light source)—(**b**).

An example of data spectra in PRIME technology in an electric circuit without any interference introduced by the operation of non-linear receivers is presented in Figure 14a. The visible voltage peak with a frequency of about 20 kHz is a parasitic interference introduced from a non-galvanically isolated city network, most likely due to electric wheeled transportation. In the case of the operation of non-linear receivers, the PLC transmission in the PRIME technology may be deteriorated. This can be seen from the comparison of the measured data spectra shown in Figure 14a,b, respectively.

As research has shown, PLC transmission in the PRIME technology can deteriorate under the operation of non-linear receivers. The nature of the spectrum deterioration depends on the type of the load, and particularly, fluorescent lamps (CFL) have indicated a significant influence among the tested receivers. The developed interference detector fully confirmed its usefulness for measuring the conducted disturbances in the 3–100 kHz band caused by the operation of non-linear receivers and, thus, for controlling the deterioration of narrow-band PLC transmission, including the PRIME technology.

## 5. Conclusions

The article presents and discusses the structure and operational parameters of the newly developed interference detector (ILD) with a specially selected Rogowski coil made with high-density printed circuit board technology (PCB HDI). This device is designed for the e-management of detection and measurement of conducted interferences (mainly related to distortion of current waveforms) that deteriorate narrow-band PLC transmission in the frequency range of 2–150 kHz. Interference in this frequency band (called supraharmonics) is mainly caused by any digital (non-linear) electronic device working in impulse mode and/or by using a switching power supply. The conducted research has shown that the majority of non-linear receivers that deteriorate the quality of electricity transmitted in the network are also a source of SH. Taking into account the fact that the developed ILD is intended for use both in low- and medium-voltage electrical networks, the parameters and dimensions of the Rogowski coil have been appropriately adapted so that the coil could be applied to electrical wires with a diameter of up to 0.05 m while ensuring a transformation ratio of not less than 1 mV/A (for 50 Hz). The validation carried out for current signals with values from 0.1 to 50 mA showed that the developed detector correctly detects and records the values and changes of current signals in the frequency range from 1 to 100 kHz. The sensitivity of the measurements was found to increase (for currents over 0.1 mA) with the interfering frequency, which is beneficial for SH detection. For user convenience, the spectrum can be displayed on a PC screen in both voltage and current values after taking into account the transformation ratio of the Rogowski coil. The conducted research confirmed the deterioration of PLC transmission in the PRIME technology by quite commonly used non-linear receivers. Fluorescent lamps (CFLs) play a significant role here. Therefore, the usefulness of the developed ILD for control of the disturbances due to SH as well as for control of the efficiency of narrow-band PLC transmission, including PRIME technology, has been demonstrated and proved.

## Data Availability

The data presented in this study are available on request from the corresponding author. The data are not publicly available due to secrecy restrictions.
